# Aquatic Organisms in Response to Salinity Stress: Ecological Impacts, Adaptive Mechanisms, and Resilience Strategies

**DOI:** 10.3390/biology14060667

**Published:** 2025-06-09

**Authors:** Tariq Dildar, Wenxiao Cui, Mhd Ikhwanuddin, Hongyu Ma

**Affiliations:** 1 Guangdong Provincial Key Laboratory of Marine Biotechnology, Shantou University, Shantou 515063, China; 21tariq@stu.edu.cn (T.D.); wxcui@stu.edu.cn (W.C.); 2 International Joint Research Center for the Development and Utilization of Important Mariculture Varieties Surrounding the South China Sea Region, Shantou University, Shantou 515063, China; ikhwanuddin@umt.edu.my; 3 STU-UMT Joint Shellfish Research Laboratory, Shantou University, Shantou 515063, China; 4 Higher Institute Centre of Excellence (HICoE), Institute of Tropical Aquaculture and Fisheries, Universiti Malaysia Terengganu, Kuala Nerus 21030, Terengganu, Malaysia

**Keywords:** salinity stress, adaptive mechanisms, aquatic organisms, osmoregulation, microbiome interaction, genetic diversity

## Abstract

Salinity stress presents a major ecological challenge for aquatic organisms, particularly in environments where salinity levels fluctuate. These fluctuations are becoming more pronounced due to climate change, further destabilizing aquatic ecosystems. Understanding how organisms adapt to such variability is essential for biodiversity conservation and the sustainable management of aquatic resources. This review examines the physiological, molecular, and behavioral adaptations that enable aquatic organisms to survive and thrive under salinity stress. Specifically, it explores mechanisms of osmotic regulation, ion transport, and oxidative stress responses, highlighting key signaling pathways—such as AMP-activated protein kinase (AMPK), Phosphatidylinositol 3-kinase–protein kinase (PI3K-AKT), Mitogen-activated protein kinase (MAPK), and the Hippo pathway—that facilitate these adaptive processes. The review also emphasizes the genetic and epigenetic modifications that contribute to resilience, underscoring the importance of genetic diversity for species survival in fluctuating salinity conditions. Furthermore, the interactions between host organisms and their microbiomes are discussed as critical factors influencing resilience. The review addresses the impact of salinity fluctuations on species distribution and biodiversity, with a focus on the implications of climate change for aquatic ecosystems. Finally, strategies for mitigating salinity stress, such as nutritional interventions and the development of salinity-resistant varieties, are explored, particularly in aquaculture. Overall, this review consolidates current knowledge on organismal adaptations, molecular mechanisms, and environmental challenges, offering valuable insights for ecological research and aquaculture practices in the face of climate change.

## Simple Summary

In aquatic ecosystems, abiotic factors, such as temperature, pH, and salinity, play crucial roles in shaping biological communities. Compared to temperature and pH, salinity is particularly significant because of its profound impact on the osmoregulatory capacities of organisms across a variety of aquatic habitats, ranging from freshwater to seawater. Understanding how organisms adapt to salinity variability is essential for biodiversity conservation and the sustainable management of aquatic resources. This review first examines the physiological, molecular, and behavioral adaptations of aquatic organisms in response to survive and thrive under salinity stress. Then, the mechanisms of osmotic regulation, ion transport, and oxidative stress responses, especially key signaling pathways involved in adaptive processes, are explored. Moreover, genetic and epigenetic modifications and the interactions between organisms and microbiomes are emphasized for their contribution to resilience. Finally, the review suggests potential strategies for mitigating salinity stress, such as nutritional interventions and the development of salinity-resistant varieties in aquaculture.

## 1. Introduction

In aquatic ecosystems, both the mean and variance of several abiotic factors, such as temperature, pH, and salinity, play crucial roles in shaping biological communities [1]. While temperature and pH are recognized as key abiotic factors influencing aquatic environments, salinity is particularly significant due to its profound impact on species’ osmoregulatory capacities across a variety of aquatic habitats, ranging from freshwater to hypersaline ecosystems [2]. Climate change is exacerbating these salinity concerns by elevating temperatures and altering precipitation patterns, resulting in more intense and unpredictable salinity variations in both freshwater and marine systems [3]. Organisms’ ability to tolerate specific salinity levels is intrinsically linked to their osmoregulatory mechanisms, which are shaped by their life history traits. To maintain osmotic balance, organisms expend considerable energy regulating ion uptake and secretion in response to hypoosmotic and hyperosmotic conditions, thereby ensuring stable internal fluid concentrations [4]. In hypoosmotic environments, organisms lose ions to the surrounding water. To maintain osmotic balance, ion transporters such as Na^+^/K^+^-ATPase (NKA) and Na^+^/K^+^/2Cl^−^ co-transporters (NKCC1) actively expel Na^+^ and retain K^+^ In contrast, in hyperosmotic environments, organisms must avoid excessive ion buildup. In this case, NKCC1-mediated Na^+^ inflow is increased to support ion absorption, whereas NKA promotes the extrusion of surplus Na^+^ to maintain homeostasis [5]. These energy-dependent systems are necessary for organisms’ survival in environments with changing salinity, ensuring that cells can adjust to the osmotic stress imposed by the surrounding medium. However, climate-driven salinity fluctuations are pushing these adaptive systems to their limits, as indicated by increased metabolic and oxidative stress in aquatic species exposed to rapidly changing salinity [6].

Despite the growing understanding of the impact of salinity on aquatic organisms, a significant research gap remains regarding the species-specific mechanisms of osmoregulation across diverse environments, particularly in the context of rapidly changing climate conditions. This study addresses this gap by exploring the adaptive strategies employed by various species to cope with fluctuating salinity levels and the broader implications of these adaptations for ecosystem stability and biodiversity. Salinity shifts can change species distributions by modifying organisms’ physiological and behavioral attributes, which influence dynamics of competition, predator–prey relationships, and community structure [7]. These changes can have a cascading effect on ecological processes like nutrient cycling, food web interactions, and habitat availability, eventually influencing aquatic ecosystem stability and function [8].

The evolution of osmoregulatory mechanisms has enabled aquatic organisms to colonize diverse ecological niches [9], with adaptations occurring at the molecular, physiological, ecological, and behavioral levels to cope with varying salinity conditions [10]. At the behavioral level, organisms may modify their feeding or movement patterns to avoid stressful conditions or seek more favorable environments. At the molecular level, they respond to salinity stress through changes in gene expression, activation of signaling pathways, and epigenetic modifications [11]. Physiologically, osmoregulatory processes involving ion transport are essential for maintaining internal homeostasis across environmental salinity gradients [3].

For species to effectively adapt to salinity stress, they must exhibit appropriate genetic diversity and population structure [12]. For instance, organisms with greater genetic diversity typically exhibit a wider range of physiological responses to fluctuations in salinity, enhancing their survival in environments with variable salinity levels [13]. Furthermore, genetic diversity in gill function, osmoregulatory processes, and the production of antioxidant enzymes plays a critical role in determining species’ ability to cope with salt-induced oxidative stress [14]. Beyond intrinsic traits, interactions between host organisms and their microbiomes can also influence their tolerance to salinity stress [15]. Specifically, by modulating host physiology [16] and enhancing stress responses [17], the microbiome can significantly affect how aquatic organisms respond to fluctuating salinity levels.

Despite the recognized importance of salinity adaptation for the survival of aquatic species, significant gaps remain in our understanding of the ecological impacts, adaptive mechanisms, and molecular responses of aquatic organisms, particularly among crustaceans and fish. This knowledge is increasingly critical in the context of global environmental changes, as anthropogenic activities not only alter salinity levels and introduce other stressors [8] but also affect the population and genetic structures of species, which in turn influence their capacity to respond to these changes [18]. This review aims to address these gaps by understanding species-specific mechanisms of osmoregulation under fluctuating salinity stress, with a particular focus on aquatic organisms such as crustaceans and fish. While existing research has explored the impact of salinity on marine life, the roles of microbiomes and genetic diversity in enhancing salinity tolerance have been insufficiently studied. Further, our objective is to investigate how adaptive strategies at the molecular, physiological, and behavioral levels contribute to survival in environments characterized by fluctuating salinity. Additionally, we aim to explore the potential role of epigenetic modifications and the complex interactions between host organisms and their microbiomes in coping with salinity stress. In addition, this study examines innovative strategies for mitigating salinity stress in aquatic organisms, such as nutritional interventions and selective breeding programs designed to enhance salinity tolerance.

## 2. Ecological Consequences of Salinity Stress on Aquatic Organisms

Aquatic organisms have developed a range of adaptive strategies to cope with the ecological pressures imposed by salinity stress, which allows them to thrive in environments with fluctuating salinity levels.

### 2.1. Salinity Ranges in Aquatic Environment

Aquatic ecosystems exhibit a wide range of salinity levels, from less than 1 ppt in freshwater environments to over 400 ppt in hypersaline habitats [3]. In estuarine regions, where freshwater meets seawater, salinity fluctuates from 0.5 to 35 ppt on a daily or seasonal basis, influenced by factors such as tides, river inflows, and evaporation [19]. These dynamic salinity gradients create distinct ecological zones, with transitional areas that support both freshwater and marine species. These zones often exhibit greater biodiversity compared to strictly freshwater or marine systems [20]. Hypersaline environments, such as shallow lagoons and arid coastal regions, may reach salinities of up to 350 ppt, as seen in areas like the Gulf of Mexico, Mediterranean, and Red Sea [21]. These lakes are renowned for their hypersaline conditions, which significantly influence the physiological responses of the organisms inhabiting them. Characterized by high concentrations of salts like sodium chloride (NaCl), sodium sulfate (Na_2_SO_4_), and other soluble minerals, these hypersaline lakes present unique challenges to the resident biota. For instance, in the Aral Sea, increasing salinity, particularly from sodium sulfate as the lake shrinks, has altered the ionic composition of the water, impacting the osmoregulatory processes of aquatic organisms [22]. Similarly, the Dead Sea, with its extreme salinity, primarily composed of chloride salts, supports only specialized extremophiles such as *Artemia salina* and other halophilic species. These organisms must adapt to high concentrations of chloride and sulfate ions, requiring significant physiological modifications in metabolic and ion-transport mechanisms to maintain homeostasis. Furthermore, the chemical composition of these hypersaline lakes, especially the dominance of chloride and sulfate ions, affects the bioavailability of essential nutrients, influencing ion exchange and osmotic regulation. Studies on the Dead Sea and similar hypersaline environments highlight how these extreme conditions drive adaptations at the biochemical and cellular levels, such as alterations in ion-transport and osmoregulatory strategies, which are critical for the survival of organisms in these challenging environments [23].

The significance of these ecological regions depends on the complex interactions between salinity and other environmental factors such as temperature, oxygen, and nutrient availability. For example, salinity-induced stratification in coastal ecosystems can lead to the emergence of separate layers in the water column, influencing the distribution of species and their access to nutrients [24]. These layers, controlled by freshwater influxes and melting ice, provide niches where certain species can thrive, while others may struggle to survive [25]. Furthermore, salinity changes shape ecotones, which are transition zones between various biological communities, such as those found at the freshwater–saltwater ecosystem interface [26]. These ecotones are centers of biodiversity, with a wide range of species, including euryhaline and stenohaline organisms. Euryhaline species, such as *Mozambique tilapia*, are particularly adapted to a wide range of salinities, allowing them to thrive in environments with frequent salinity changes [27]. These species perform a vital role in maintaining ecological stability by enabling species migration and fostering biodiversity in transitional conditions [28]. In contrast, stenohaline species, such as *Cyprinus carpio*, are confined to stable environments with narrow salinity ranges and are more vulnerable to salinity changes in transitional zones [29].

### 2.2. Effect of Salinity Variability on Species Distribution and Biodiversity

The effect of salinity variability on species distribution and biodiversity in estuarine ecosystems is significant, as species exhibit varying levels of tolerance to fluctuating salinity levels. Euryhaline species, capable of thriving across a broad range of salinity conditions, contrast with stenohaline species, which are restricted to either freshwater or marine environments with stable salinity. Fluctuations in salinity can induce physiological stress, particularly in invertebrates, which typically show less tolerance compared to fish [30]. Such stress can drive significant shifts in community composition, especially within invertebrate populations, as many species are replaced by lentic taxa better adapted to rising salinity [31].

Extreme cases of salinity fluctuation are found in hypersaline environments, where salinity levels exceed that of seawater. These environments tend to exhibit reduced biodiversity due to their harsh conditions. However, some species have evolved remarkable adaptations to survive in hypersaline conditions. For example, *Artemia* can tolerate salinities exceeding 100 ppt, utilizing advanced osmotic regulatory mechanisms [32]. On the other hand, *Oreochromis mossambicus* and *Oreochromis niloticus* reveal significant tolerance to varying salinities, with the ability to adapt to salinity levels of 0–100 ppt and 0–35 ppt, respectively. In these extreme environments, species richness tends to decline, and community composition shifts toward a small number of highly specialized species [33,34]. As salinity surpasses 35 g/L, only species with exceptional salinity tolerance are able to persist, resulting in the dominance of crustaceans, particularly *Artemia*, which is the most prevalent taxon in these ecosystems [35].

The dominance of specialized species in hypersaline environments underscores the remarkable adaptability of organisms to fluctuating salinity levels. These adaptations are critical for maintaining ecosystem functions such as nutrient cycling and food web dynamics [36]. For example, in hypersaline ecosystems, halophilic invertebrates like *Artemia* thrive at extreme salinities (>120–160 g/L), driving efficient energy transfer from primary production to higher trophic levels, despite the absence of higher consumers [37]. These simplified food webs consist primarily of *Artemia*, and the absence of complex predation pressures results in cascading effects, where *Artemia* reduces phytoplankton populations, significantly altering nutrient cycling and primary production [38]. Vertebrates such as salt-tolerant fish (up to 120 g/L) and specialist waterbirds also influence ecosystem functioning; flamingos and other birds reduce *Artemia* numbers while spreading nutrients across the landscape [39]. This results in a dynamic interplay between bottom-up (invertebrate-mediated) and top-down (avian predation) mechanisms that preserves the particular productivity of hypersaline ecosystems despite their lower biodiversity [39,40].

### 2.3. Climate Change Implications on Salinity Fluctuations

Climate change is altering salinity dynamics in marine ecosystems, primarily through increased evaporation, shifting precipitation patterns, and polar ice melt, all of which contribute to both hyper- and hyposaline conditions [41]. These salinity fluctuations interact with other climate stressors, such as ocean warming and acidification, creating complex challenges for marine organisms. For instance, marine bivalves like *Crassostrea gigas* and *Mytilus edulis* exhibit increased carbonic anhydrase activity under conditions of both low salinity and ocean acidification, suggesting potential negative impacts on biomineralization and shell formation. The compounding effects are particularly severe when temperature and salinity stressors coincide, as seen in mussels that exhibit elevated expression of stress-related genes under both low salinity and high temperature conditions [42].

The physiological impacts of these combined stressors are leading to concern ecological consequences. Some species facing simultaneous temperature increases and salinity fluctuations surpass their physiological tolerance thresholds, potentially leading to decreased fitness, reproductive failure, and increased mortality rates [43]. Freshwater species are especially vulnerable to salinization effects, with studies documenting significant physiological stress responses in fish populations. Geographic spawners face particular challenges, as decreasing stream flows and higher temperatures disrupt their reproductive cycles, sometimes leading to complete spawning failures [44]. These climate-driven changes are further complicated by ocean acidification, which affects behaviors and survival rates in fish larvae [45].

Climate-induced shifts are also driving significant changes in species distributions, leading to disease outbreaks in several species and having wide-ranging impacts on ecosystem dynamics [46]. For example, warming water above 15 °C impairs the immune system of *Homarus americanus* (temperate North Atlantic), making them more susceptible to diseases like gaffkemia and epizootic shell disease. In *Chionoecetes opilio* (Arctic and sub-Arctic regions), thermal stress has triggered outbreaks of bitter crab disease caused by Hematodinium, with prevalence increasing five-fold per 1°C rise in temperature [47]. These shifts disrupt host–pathogen dynamics, often favoring pathogen proliferation over host defenses, particularly in ectothermic species.

To address these challenges, adaptive strategies for management are essential for aquaculture systems. Selective breeding programs focus on developing climate-resilient strains, including salinity-tolerant species [48,49]. Nutrigenomic approaches offer potential for mitigating stress impacts through customized dietary formulations [49], while advanced reproductive technologies like surrogate broodstock help preserve genetic diversity under changing conditions [50].

## 3. Organismal Adaptations to Salinity Stress

To persist and thrive in environments with variable salinity, aquatic organisms employ a range of adaptive strategies that include both physiological and behavioral mechanisms. This section explores these adaptations in detail, examining the physiological, behavioral, molecular, and cellular mechanisms that enable these organisms to withstand and adapt to such fluctuations.

### 3.1. Adaptive Strategies for Salinity Tolerance in Aquatic Organisms

Maintaining osmotic homeostasis in environments where salinity fluctuates is crucial for survival. Freshwater species, such as teleost fish, maintain osmotic balance by actively absorbing salt from the environment and excreting diluted urine to counteract passive salt loss and excessive water influx. In contrast, marine species face the challenge of preventing dehydration and excessive salt accumulation. These species conserve water and excrete excess salts primarily through specialized cells in the gills and kidneys [51].

Euryhaline organisms use complex molecular processes to maintain osmotic balance under changing salinity conditions, with aquaporins (AQPs) and ion transporters playing crucial functions in cellular water and ion homeostasis. Euryhaline organisms, such as *O. niloticus*, use similar osmoregulatory systems, such as NKA, to control ion transport and maintain ionic balance. *O. niloticus* can modify their osmoregulatory functions to changing salinities by depending on ion transporters and aquaporins to manage water and ion homeostasis, which is essential for survival in both freshwater and brackish environments [52]. Studies on crustaceans show that AQPs (AQP3, AQP4, and AQP11) are differentially expressed in osmoregulatory organs such as gills, where they control water permeability to prevent excessive efflux during high-salinity stress. These proteins collaborate with ion-transport genes including NKAα, carbonic anhydrase, and V-type H^+^-ATPase (VHA) subunits to control ion uptake and excretion. For example, in *Fenneropenaeus chinensis*, coordinated overexpression of AQP3/4 and ion transporters in the gills improves salinity tolerance by regulating cellular hydration and ionic balance [53]. Similarly, *Litopenaeus vannamei* has tissue-specific AQP expression patterns, with acute salinity changes inducing dynamic alterations in AQP3, AQP4, and AQP11 levels to improve water and ion transport [54]. These molecular adaptations enhance physiological mechanisms such as *Macrobrachium* spp. spawning in brackish water and energy-efficient ion regulation mediated by NKA and VHA, allowing survival over an extensive range of saline conditions [55].

Salinity tolerance in *Oreochromis niloticus* (Nile tilapia) is a crucial factor for expanding its aquaculture potential in brackish and saline environments. Recent studies have revealed that Nile tilapia exhibits significant physiological adaptations to higher salinity, particularly in its gill structure and osmoregulatory mechanisms. The proliferation of chloride cells and increased Na^+^/K^+^ ATPase activity in the gills enable these fish to better regulate ion balance in saline conditions [52]. Additionally, studies on the natural salinity tolerance of different *O. niloticus* haplotypes indicate that selecting strains with inherent salinity tolerance could further optimize aquaculture systems in coastal and brackish water regions [56]. While high salinity can negatively affect reproduction, limiting population growth, this characteristic could be utilized as a population control strategy in aquaculture [43]. Additionally, studies show that proper acclimatization and dietary management can further optimize growth in saline conditions, with brackish water (5–10 ppt) being particularly suitable for farming Nile tilapia in intensive systems [57].

Diadromous fish, which migrate between freshwater and marine environments, exhibit complex life cycles that accommodate transitions between distinct salinities. Their ability to maintain euryhalinity has evolved independently across multiple teleost lineages, demonstrating the remarkable physiological and evolutionary flexibility of these species [58].

Marine elasmobranchs employ a distinct osmoregulatory strategy, using organic osmolytes such as trimethylamine-N-oxide (TMAO) to maintain osmotic balance. They excrete excess NaCl through the rectal gland, a strategy that contrasts with most teleost fish, which rely on different osmoregulatory mechanisms to actively regulate their extracellular fluid osmolality [59]. In contrast, hagfish are osmoconformers, maintaining body fluid NaCl concentrations that closely match seawater, thereby minimizing the need for active osmoregulation [60]. These varied strategies highlight the diverse adaptations among fish species to maintain osmotic balance across different environments.

Crustaceans have evolved isosmotic intracellular regulation, a mechanism that allows cells to adjust their volume in response to osmotic stress. In low salinity environments, cells reduce their regulatory volume to prevent swelling, while in high-salinity environments, they increase their regulatory volume to maintain cellular integrity. This intracellular regulation is favored over isosmotic extracellular regulation due to its greater efficiency in managing cellular volume under varying salinity conditions [55]. Additionally, osmoregulatory capacities can be gender-specific, as seen in species like *Callinectes sapidus*, where such mechanisms may contribute to the species’ invasive success [61].

### 3.2. Behavioral Adaptations to Salinity Fluctuations in Aquatic Organisms

Aquatic organisms living in ecosystems with frequent salinity fluctuations have developed a variety of behavioral adaptations to cope with these changes. A key strategy is selective habitat use. Many species, particularly those in hypersaline or brackish environments, avoid high-salinity areas by seeking out habitats with lower salinity, thus minimizing osmoregulatory stress. This behavior enables them to allocate energy toward fitness enhancing functions [62]. Studies show that different species use different behavioral strategies. For instance, the euryhaline *Kryptolebias marmoratus* increases activity in hypersaline conditions to find lower salinity refuges, whereas *Danio rerio* exhibit significantly reduced locomotor activity at high salinity (5.78 g/L), conserving energy by reducing movement duration and distance traveled during both light and dark phases [63]. Interestingly, *Poecilia latipinna* exhibit more varied responses that are not precisely related to salinity concentration, implying species-specific adaptive processes. Additionally, *P. latipinna* prefers lower salinity habitats, especially when subjected to additional stresses such as predator cues [64]. This variability in tolerance enhances the overall population’s ability to survive and thrive under fluctuating environmental conditions.

### 3.3. Osmotic Stress and Ion-Transport Mechanism Response in Fluctuating Salinity Environments

**Figure 1:**
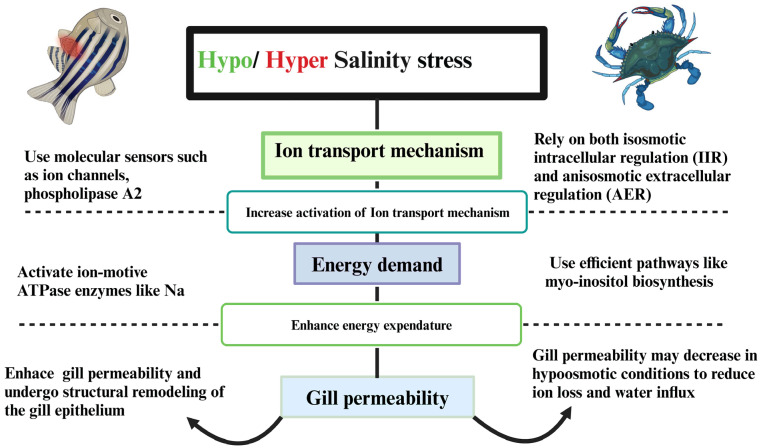
The physiological responses of aquatic organisms to hypo- and hyper-salinity stress, highlighting the activation of ion-transport mechanisms to maintain osmotic balance. This increased ion transport leads to higher energy expenditure, which in turn affects gill permeability, influencing the overall physiological response of aquatic organisms to fluctuating salinities.

The physiological response of aquatic organisms to hypo- and hyper-salinity stress emphasizes the complex mechanisms involved in maintaining osmotic balance. When exposed to fluctuating salinity, organisms activate ion-transport mechanisms, such as Na^+^/K^+^-ATPase, to regulate the movement of ions across their membranes and maintain osmotic equilibrium. This activation comes at a significant cost, however, as it increases the energy demand required to support enhanced transport. To meet these heightened energy requirements, organisms must increase their overall energy expenditure. Additionally, as part of this adaptive response, gill permeability is adjusted to facilitate more efficient ion exchange. Collectively, these physiological adjustments enable organisms to cope with the challenges posed by fluctuating salinity environments, ensuring their survival and maintaining homeostasis under osmotic stress (Figure 1) [3].

Exposure to hyperosmotic conditions triggers specific molecular responses to support osmotic regulation. For example, hyperosmotic stress activates phosphorylation of tight junction proteins by myosin light chain kinase, which modulates the activity of Na^+^/Cl^−^/taurine co-transporters, helping to maintain ion balance [65]. In hypoosmotic conditions, dephosphorylation of focal adhesion kinase enhances the activity of Na^+^/K^+^/2Cl^−^ co-transporters and Cystic Fibrosis Transmembrane Conductance Regulator, aiding ion regulation and restoring osmotic equilibrium [66]. Furthermore, the expression of osmotic stress transcription factor 1 Osteoclast Stimulating Factor 1 is upregulated in response to salinity fluctuations, further modulating these transport mechanisms to help organisms to adopt varying osmotic environments [67].

Different species exhibit unique adaptations to cope with salinity fluctuations. For example, *Sinonovacula constricta* can maintains osmotic balance by modulating free amino acids like taurine and increasing Na^+^/K^+^-ATPase activity in the gills under fluctuating salinities [68]. Similarly, juvenile *Eriocheir sinensis* display optimal osmoregulatory performance at 8‰ salinity, but their ion transport is disrupted when salinity deviates from range [69].

Some marine fish also exhibit remarkable adaptations to fluctuating salinity. Species such as *O. mossambicus* undergo extensive gill remodeling and adjust their ion-transport systems to meet the high energy demands of fluctuating salinities [70]. Similarly, the *Fundulus heteroclitus* and Tilapia sp. adjust ion transport and use neuroendocrine regulation to cope with salinity changes [71]. The *Acanthopagrus schlegelii* also adapts by changing gene expression in its gills, enhancing ion-transport efficiency and adjusting Na^+^/K^+^-ATPase activity [72].

Chronic salinity stress requires long-term metabolic adjustments to maintain homeostasis. For example, in response to prolonged salinity exposure, *Salmo salar* undergo metabolic reprogramming that regulates enzymes and pathways for salt metabolism to cope with prolonged salinity exposure [51]. In fish, osmoregulation accounts for 1–50% of total energy expenditure, which increases during extreme salinity stress [73]. Brine shrimp (*Artemia*) are metabolically stable around 35–150 g/L but consume more oxygen below 35 g/L, indicating increased osmoregulatory needs [74]. Organisms maintain homeostasis by implementing metabolic changes, such as switching energy substrates [75] or increasing ion transporters like Na^+^/K^+^-ATPase to enhance efficiency [76].

### 3.4. Oxidative Stress Response in Aquatic Organisms Due to Salinity Stress

Salinity stress induces oxidative stress by disrupting redox balance through interactions between osmotic and oxidative pathways (Table 1) [77]. This imbalance increases the production of reactive oxygen species (ROS), leading to cellular damage. The production of ROS occurs as a result of altered cellular respiration and metabolic processes in response to salinity changes [3]. For example, when aquatic organisms experience osmotic stress, this disrupts the balance between the generation and scavenging of ROS [78]. In cells, changes in ion concentrations (such as sodium and chloride ions) can affect mitochondrial function, leading to an increase in electron leakage during cellular respiration. This electron leakage can result in the production of superoxide anions (O^2•−^), a primary ROS [79].

To counter this oxidative stress, organisms activate antioxidant defense mechanisms such as SOD, catalase (CAT), and glutathione peroxidase (GPx). These antioxidants work in concert to neutralize ROS. For instance, SOD catalyzes the dismutation of superoxide into oxygen and hydrogen peroxide, while CAT converts hydrogen peroxide into water and oxygen, and GPx reduces hydrogen peroxide or lipid peroxides to water or corresponding alcohols, respectively [80]. In some species, increased ROS production due to osmotic stress can also lead to alterations in the expression of these antioxidant enzymes.

For example, fluctuating salinities elevate ROS levels in *Scylla serrata*, which triggers the activities of these antioxidant enzymes, such as SOD and CAT, to reduce oxidative damage [81]. On the other hand, rapid salinity reductions in *E. sinensis* compromise oxidative balance by lowering antioxidant enzyme activities, but enhance defenses through an increase in total antioxidant capacity (T-AOC), GSH-PX, and CAT activity [82].

**Table 1:** Salinity stress-related changes in antioxidant enzyme activity across different aquatic species.

Species	Salinity Type	Duration	Enzyme Activity	References
*Brachionus koreanus*	High	3–24 h	ROS and GST activity elevated	[83]
*Crassostrea gigas*	High/Low	4 months	Elevated level of CAT, whereas no effect observed at SOD	[84]
*Litopenaeus vannamei*	High/Low	24 h	Activities of SOD, GPx, and CAT decreased at both lower and higher salinities	[85]
*Eriocheir sinensis*	High/Low	24–72 h	At higher salinities, GSH-Px activity is enhanced; works synergistically with SOD and CAT to mitigate lipid peroxidation damage	[86]
*Procambarus clarkiis*	Low	48 h	Antioxidants like CAT and GPX-PX decrease, while MDA levels increase	[87]
*Scylla olivacea*	Medium	Varied	Increase in gill SOD activities helps cope with elevated ROS levels from high metabolic demands	[88]
*Lates calcarifer*	High/Low	8 weeks	GPX and CAT activities increase in high salinity and decrease in low salinity	[89]
*Oreochromis niloticus*	Medium/Low	4 weeks	The activity of SOD, GPX, and CAT elevates under medium salinity alongside hypoxia	[90]
*Dicentrarchus labrax, Chanos chanos*	High/Low	4 weeks	SOD level of activity elevated in both fresh and saline water	[91]
*Cyprinus carpio*	Medium/Low	8 weeks	Exposure to medium salinity reduced lysozyme activity, SOD, CAT, GPx, and increased MDA levels before and after heat stress	[92]

Species can adapt to salinity-induced oxidative stress by altering respiration and metabolic strategies. For instance, *Tigriopus brevicornis* reduces oxygen consumption under stressful conditions, suggesting a different metabolic strategy to conserve energy and mitigate oxidative damage [93]. In bivalves such as *C. gigas*, the activation of antioxidant enzymes is triggered, which helps mitigate oxidative stress [84]. The sensitivity of *Mytilus galloprovincialis* to oxidative stress is particularly evident when salinity fluctuates. At low salinity, markers like protein carbonyls and Thiobarbituric acid reactive substances significantly increase in gill tissues, indicating oxidative stress and cellular damage in *M. galloprovincialis* under fluctuating salinities [94]. These studies support the dynamic nature of oxidative stress responses in bivalves under varying salinity conditions.

Fish also employ diverse strategies to manage oxidative stress caused by salinity fluctuations. For example, *Chanos chanos* upregulate enzymatic antioxidants to maintain redox balance and prevent apoptosis in seawater, but in freshwater, despite the compensatory increase in antioxidants, *C. chanos* still undergoes apoptosis due to an imbalance between ROS production and the capacity of antioxidant defenses to neutralize the oxidative stress [91]. Similarly, antioxidant-rich diets further enhance these enzymatic defenses, as observed in *Salmo trutta*, where dietary supplements improve CAT and GPx activity, thus protecting against oxidative damage [95]. According to these studies, fish employ a range of strategies, including enzymatic regulation and dietary adaptations to mitigate oxidative stress and maintain redox homeostasis under fluctuating salinity conditions.

### 3.5. Molecular Responses to Salinity Stress

#### 3.5.1. Key Signaling Pathways

**Figure 2:**
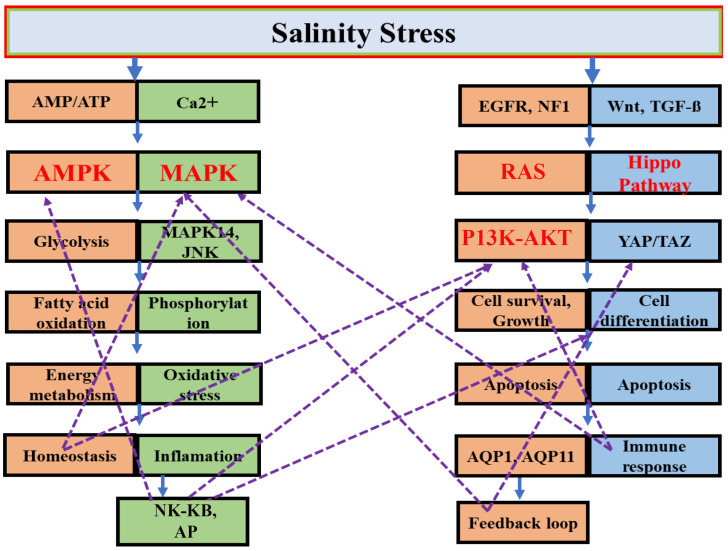
Salinity stress activates the MAPK, RAS, PI3K-AKT, AMPK, and Hippo pathways. In this figure, the red color represents the pathways, highlighting their roles in the stress response, while other colors correspond to the responses related to these pathways. The MAPK pathway induces stress and inflammatory responses through proteins such as MAPK11, JNK, and MKK4. The RAS and PI3K-AKT pathways elevate cell survival and osmoregulation. The AMPK pathway is triggered by AMP/ATP ratio alterations, which improves energy metabolism. The Hippo pathway, in conjunction with YAP/TAZ, modulates cell proliferation and immunological responses.

Signaling pathways are vital in mediating responses to environmental stressors like salinity fluctuations. These pathways regulate several important processes, including ion transport, osmoregulation, cell survival, metabolism, and immunity. This section examines important signaling pathways—AMPK, PI3K-AKT, MAPK, and the Hippo pathway (Table 2)—that play a fundamental role in maintaining homeostasis. The intricate molecular signaling pathways activated in response to salinity stress highlight the complex mechanisms that enable organisms to cope with fluctuating salinity levels. Upon exposure to salinity stress, two major pathways, AMPK and MAPK, are activated to regulate critical processes such as glycolysis, fatty acid oxidation, and energy metabolism, thereby maintaining cellular homeostasis. Additionally, calcium (Ca^2+^) signaling plays a pivotal role in triggering intracellular responses. The figure also emphasizes the involvement of the RAS, P13K-AKT, and YAP/TAZ pathways, which are essential for regulating cell survival, growth, apoptosis, and differentiation under stress. These signaling cascades interconnect in a feedback loop, ensuring a coordinated response. Furthermore, the figure underscores the importance of aquaporins (AQP1 and AQP11) in managing osmotic balance by controlling water and ion transport. Immune responses, inflammation, and apoptosis are also regulated by these pathways to mitigate cellular damage. Collectively, this network of molecular responses allows organisms to adapt to salinity fluctuations, ensuring their survival and maintaining cellular integrity under osmotic stress (Figure 2).

##### MAPK Signaling Pathway Response in Aquatic Species Under Salinity Stress

The mitogen-activated protein kinase (MAPK) signaling pathway regulates response to salinity stress by transmitting environmental signals to the nucleus for adaptive changes [106,107]. Salinity-induced increases in cytosolic calcium (Ca^2+^) activate the MAPK pathway, one of several signaling pathways responding to osmotic stress. This activation leads to the phosphorylation of key proteins involved in stress responses, including transcription factors and enzymes that help the organism adapt to changes in salinity [108]. For example, in the *Scophthalmus maximus*, p38 MAPK upregulation induces downstream phosphorylation events that mediate osmoregulation [106]. Similarly, in *Lateolabrax maculatus* hyperosmotic shock triggers MAPK11 (p38β) expression, further supporting the MAPK pathway role in salinity stress responses [109]. Additionally, MAPK14a has been associated to oxidative damage in *Ictalurus punctatus* under extreme salinity stress, highlighting the role of MAPK pathways in managing both osmotic and oxidative stress [98].

Among MAPK subfamilies, c-Jun N-terminal kinase (JNK) plays a prominent role in managing osmotic and oxidative stress, as well as regulating metabolism, inflammation, and apoptosis. For instance, in *L. maculatus*, JNK activation facilitates osmotic adaptation by regulating mitogen-activated protein kinase 4 (MKK4) [109]. A similar mechanism is observed in the fish *Rachycentron canadum*, where extreme salinity fluctuations lead to changes in MAPK gene expression, particularly in the gills and kidneys, underscoring their involvement in ion transport and osmotic regulation during low salinity stress [97].

In addition to osmotic stress, salinity combined with elevated carbonate alkalinity activates the MAPK pathway, leading to increased ROS production and apoptosis in *E. sinensis* [110]. Similarly, in *Penaeus monodon*, JNK activation regulates MKK4 during low-salinity adaptation [111]. In *Chlamys farreri*, JNK expression increases sharply within hours of environmental stress, highlighting the rapid role of JNK in molluscan stress responses [98].

##### PI3K-AKT Signaling Pathways in Response to Salinity Stress

The PI3K-AKT signaling pathway, often activated by RAS, plays a vital role in physiological regulation and cellular resilience. In crustaceans, this pathway is integral not only to cellular resilience under stress but also to the innate immune response [112]. The central components of this pathway, phosphatidylinositide 3-kinases (PI3Ks) and AKT (protein kinase B), regulate essential cellular processes. For instance, they inhibit pro-apoptotic proteins, such as the BCL2-antagonist of cell death, and prevent NF- κB nuclear translocation from cells under stress [113].

In response to low salinity, the PI3K-AKT pathway modulates gene expression and protein synthesis to maintain tissue integrity and remove damaged cells through apoptosis in *S. maximus* [96]. Notably, low-salinity stress downregulates AKT1 phosphorylation and gene expression, particularly in the gill, highlighting tissue-specific responses [101]. Additionally, this pathway mediates the expression of aquaporin genes such as AQP1 and AQP11, and the PI3K-AKT pathway facilitates water and ion exchange essential for maintaining osmotic balance in fluctuating environments [114].

Brain transcriptome profiling analysis of *Oreochromis niloticus* under prolonged hypersaline stress revealed that the PI3K-AKT signaling pathway plays a key role in the upstream regulation of osmoregulatory processes in response to salinity stress [113]. Key genes within this pathway, including G protein-coupled receptors and solute carrier families, exhibit differential expression under salinity fluctuations [115]. Furthermore, inhibition of this pathway confirms its pivotal role in modulating ion channel activity, which is essential for maintaining osmotic balance under stress [101].

##### AMPK Signaling Pathway and Energy Regulation in Response to Salinity Stress

AMP-activated protein kinase helps to maintain energy homeostasis by monitoring the AMP/ATP ratio. When this ratios increase, AMPK is activated through phosphorylation at threonine 172 in the AMPKα subunit, triggering metabolic adjustments to restore energy balance [116]. Under salinity stress, AMPK modulates energy metabolism to meet the heightened demands of osmoregulation [105].

Building on this general mechanism, crustaceans exhibit unique metabolic adaptations under salinity stress. In *L. vannamei*, salinity stress alters lipid metabolism, influencing fatty acid biosynthesis and glycosphingolipid metabolism to meet energy demands [117]. In *S. paramamosain*, low salinity stress increases the AMP/ATP ratio in the hepatopancreas, thereby enhancing ATP production and upregulating genes involved in glycogen and lipid metabolism. These metabolic changes sustain energy needs during stress and facilitate osmotic pressure regulation [100].

In addition to crustaceans, fish also exhibit AMPK-mediated metabolic responses to salinity stress. In *Paralihthys olivaceus*, salinity fluctuations activate AMPK through phosphorylation and allosteric AMP binding, leading to increased glycolysis and fatty acid oxidation that provides the necessary energy to manage osmotic challenges [118]. Similarly, in *Ctenopharyngodon Idella*, activation of AMPK by 5-aminoimidazole-4-carboxamide ribonucleotide reduces serum glucose levels and regulates lipogenesis-related proteins, effectively reallocating energy resources under stress [119].

##### The Role of the Hippo Signaling Pathway in Salinity Stress

The Hippo signaling pathway plays a role in maintaining tissue and cellular integrity by integrating environmental cues and regulating cellular processes [120]. This pathway plays a role in stress responses and innate immunity in aquatic species like *L. vannamei*. Under optimal conditions, it regulates cell proliferation and anti-apoptotic mechanisms; however, during stress, such as salinity stress, the pathway shifts to promote apoptosis and cell cycle arrest to preserve cellular homeostasis [54]. In bivalves, the pathway normally promotes cell proliferation and anti-apoptotic mechanisms under optimal conditions; however, under stress, such as salinity stress, it shifts towards triggering apoptosis and cell cycle arrest to maintain cellular homeostasis [121].

Beyond its direct effects, interactions with other signaling pathways further enhance its role in managing environmental stress. Its crosstalk with the Wnt pathway, via β-catenin-mediated gene activation, supports tissue growth and repair. Further interactions with bone morphogenetic proteins and the transforming growth factor-β superfamily are vital for muscle growth and innate immunity in both crustaceans and fish [122]. The regulation of metabolism and regeneration by YAP/TAZ further emphasizes Hippo’s pivotal role in balancing apoptosis and cell survival during stress and repair. This function is vital for adaptation and resilience in stressful environments [123]. Together, these mechanisms highlight the pathway’s pivotal role in enabling adaptive responses to changing salinity conditions in aquatic organisms.

The complex interplay of these pathways regulates cellular responses to salt stress via synergistic interaction and antagonistic regulation. Under moderate salinity stress, these pathways interact to promote cell survival and adaptation. The PI3K-AKT pathway stimulates Hippo-YAP/TAZ signaling by inhibiting LATS1/2 kinases, enabling tissue repair and cell proliferation [121]. Simultaneously, MAPK (especially JNK/p38) and Hippo regulate an integrated response wherein JNK-mediated phosphorylation of YAP/TAZ increases antioxidant defenses while retaining the ability to induce apoptosis if stress becomes extreme [124]. However, under extreme salinity conditions, these pathways typically possess antagonistic interactions. AMPK activation combats PI3K-AKT signaling by suppressing mTORC1 via TSC2 phosphorylation, effectively favoring energy conservation for osmoregulation over growth activities [101]. The pathways also show tissue-specific interactions, with gill tissues preferring AMPK-PI3K synergy for ion regulation and hepatopancreatic tissues using AMPK-Hippo antagonism to manage metabolic demands [125]. The immune osmotic balance is regulated by PI3K-AKT and MAPK interaction in NF-κB activation [126]; however, this is counterbalanced by PI3K-AKT’s repression of JNK-induced apoptosis in crustaceans like *P. monodon* [127]. These dynamic interactions impose a complex regulatory network, enabling organisms to precisely regulate their stress responses based on the magnitude of salinity fluctuations.

#### 3.5.2. Stress-Responsive Genes

Stress-responsive genes play pivotal roles in regulating processes such as osmoregulation, metabolism, immune function, and apoptosis under salinity stress [128]. Heat shock proteins (HSPs), particularly HSP70 and HSP90, are molecular chaperones that stabilize cellular proteins under osmotic stress. In fish species such as *Luciobarbus capito*, high salinity triggers the expression of stress-responsive genes, including, HSP70 and HSP90 in the spleen, which helps stabilize cellular proteins under osmotic stress [129]. Transcription factors such as Forkhead box O proteins further mediate stress responses by regulating genes involved in stress resistance, metabolism, and apoptosis, enhancing adaptability during osmotic challenges [130]. In *Acanthopagrus schlegelii* the upregulation of Osteoclast Stimulating Factor 1 regulates downstream genes for ion transport and stress response [128]. Likewise, caspase genes, which are key regulators of apoptosis, are modulated by salinity stress. In *Takifugu fasciatus*, caspase 3, 7, and 9 are upregulated under high salinity, promoting apoptosis, while their expression decreases at moderate salinity, favoring cell survival [99]. Similar patterns are observed in the *Syngnathus typhle*, where salinity stress compromises immune defense [131]. Cell cycle-related genes, such as p53, are activated to promote the proliferation of ionocytes, enhancing the ion exchange capacity of gills in response to changing salinity [132]. This adjustment facilitates the organism’s immediate survival under stressful conditions.

In addition to apoptosis regulation, other genes, such as encoding aquaporins (AQPs), play a significant role in osmoregulation during salinity stress. In *L. vannamei*, the expression of LvAQP3, LvAQP4, and LvAQP11 decreases across tissues under salinity stress, except in the hepatopancreas, where they facilitate osmoregulation through amino acid metabolism [133]. Similar to the effects observed in *L. vannamei*, *Fenneropenaeus chinensis* also shows downregulation of AQP3 and AQP4 in the gills to minimize water loss and regulate ion balance [53]. These results indicate the adaptive role of AQPs in maintaining osmotic and ionic balance.

Beyond water transport, metabolic adjustments are essential for energy allocation during salinity stress. In *S. paramamosain*, enzymes like citrate synthase are upregulated to enhance energy production through the TCA cycle, while glycogen synthase and hexokinase are downregulated, indicating a shift from energy storage to immediate use [134]. Lipid metabolism also adopts accordingly, with reduced fatty acid synthesis via fatty acid synthase and increased lipid mobilization to meet energy demands [135]. Under both hypoosmotic and hyperosmotic conditions, organisms adjust the synthesis and degradation of organic osmolytes to maintain osmotic balance. Enzymes like ornithine decarboxylase are upregulated to boost the production of polyamines, while glutamine synthetase expression is downregulated, fine-tuning osmolyte metabolism and supporting cellular homeostasis [136].

#### 3.5.3. Epigenetic Modifications Under Salinity Fluctuations

**Figure 3:**
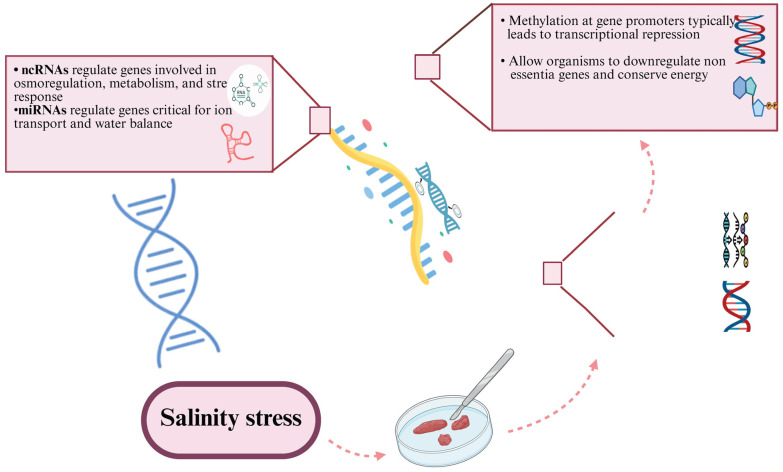
The role of non-coding RNAs (ncRNAs) and microRNAs (miRNAs) in regulating gene expression under salinity stress. It emphasizes how methylation at gene promoters leads to transcriptional repression, allowing organisms to conserve energy by downregulating non-essential genes. It also highlights the regulation of gene expression through histone modifications, affecting chromatin structure and transcription factor access to DNA.

Epigenetic modifications such as DNA methylation, histone modifications, and non-coding RNA activities enable organisms to adapt to salinity stress. These heritable molecular changes dynamically regulate gene expression, facilitating both immediate responses and long-term resilience to salinity fluctuations without altering the DNA sequence [137]. By modulating gene activity, epigenetic mechanisms help organisms cope with environmental changes in ways that are heritable and adaptive over time. Figure 3 illustrates the critical role of epigenetic modifications and non-coding RNAs (ncRNAs) in regulating gene expression in response to salinity stress. Non-coding RNAs, including microRNAs (miRNAs), regulate genes involved in osmoregulation, metabolism, and stress response, particularly those crucial for ion transport and water balance. These ncRNAs enable organisms to fine-tune gene expression in response to fluctuating salinity levels, ensuring optimal survival. Additionally, DNA methylation at gene promoters typically leads to transcriptional repression, allowing organisms to downregulate non-essential genes and conserve energy, especially under stress conditions. Histone modifications, such as the methylation of lysine residues on histone H3, further regulate gene expression by altering chromatin structure, thus controlling the accessibility of transcription factors to DNA. Together, these epigenetic mechanisms enable organisms to efficiently adapt to salinity stress, maintaining essential physiological functions while minimizing energy expenditure on non-critical processes.

In the context of salinity fluctuations, studies have shown that low salinity induces tissue-specific methylation changes in organisms. For instance, in *Portunus trituberculatus*, low salinity increases methylation in gill tissues, while decreasing it in muscle and gills [138]. Likewise, in *C. gigas*, salinity fluctuations leads to DNA methylation changes in genes associated with nucleic acid metabolism, tropomyosin, and cellular transport, highlighting the role of these modifications in osmoregulatory adaptation [139].

In addition to DNA methylation, histone modifications and non-coding RNAs also contribute to osmoregulatory responses. In *Daphnia magna*, miRNAs are involved in transgenerational inheritance of salinity tolerance, where sustained hypomethylation of stress-relevant genes persists across generations [140]. Such epigenetic memory prepares offspring for similar environmental challenges, highlighting the evolutionary significance of these mechanisms.

Several studies have reported evidence for the heritability or transgenerational transmission of epigenetic adaptation under environmental stresses [141]. However, there is little research on transgenerational inheritance under saline stress. For example, in *D. magna*, high-salinity exposure in the parental F0 generation resulted in distinct low methylation patterns in six protein-coding genes involved in DNA repair, cytoskeleton organization, and protein synthesis in the unexposed F1, F2, and F3 generations [142]. Some studies have examined transgenerational plasticity in response to salinity changes. For instance, research on the *Gasterosteus aculeatus* has investigated its adaptability to changing salinity conditions. The results show that the outcomes of transgenerational plasticity may vary depending on life stage, with early life stages being especially vulnerable to increased salinity [143]. Similarly, research on *Crassostrea virginica* evaluated transgenerational plasticity in response to low salinity. The findings revealed that transgenerational plasticity possessed no substantial impact on low-salinity tolerance, with parental genotype playing a significant role in determining larval size variation. This suggests that evolutionary adaptation is the primary strategy by which *C. virginica* can tolerate future salinity reductions. In some marine fish species, low salinity triggers distinct epigenetic response. Likewise, prolonged exposure to low salinity in *Cynoglossus semilaevis* alters H3K4me3 patterns influencing growth and liver structure. It also upregulates demethylase genes and DNA methyltransferases, enhancing methylation in renal tissues and regulating immune-related genes like *MASP1* to balance energy use and immune responses under stress [144]. These studies indicate that epigenetic modifications enable organisms to adaptively respond to environmental stressors like salinity fluctuations, ensuring both immediate and long-term survival by synchronizing physiological and metabolic processes across generations.

## 4. Microbiome Interactions and Response Under Salinity Stress

Host–microbiome interactions play a significant role in salinity tolerance as the gut microbiota play a role in metabolism, immune regulation, and disease resistance [145]. Changes in salinity can lead to shifts in the microbial community composition, often favoring opportunistic microbial taxa like *Proteobacteria* and *Vibrio* spp. under higher salinity conditions, while lower salinity levels tend to favor beneficial bacteria such as *Actinobacteria* and *Lactobacillus* [146]. These patterns are consistent across some species, as demonstrated in *Penaeus monodon*, where altered salinity destabilized gut microbial networks, elevated pathogenic potential (particularly *Vibrio*), and compromised immune functions, with 35 microbial biomarkers accurately diagnosing salinity stress [15]. Similarly, in fish species like *Gambusia* and *Paracheirodon sphenops,* salinity variations disrupted their gut microbiome, adversely impacting their health [147]. Meanwhile, gut microbiome of *Oxygymnocypris stewartia* did not show any microbiome change in response to stress [148], indicating a species-specific response of the microbiome under salinity stress.

The observed microbiome shifts under salinity stress may contribute to changes in salinity tolerance rather than being stress-induced. For example, in the case of *Nibea albiflora*, high salinity favors harmful *Vibrio* species, whereas intermediate salinity promotes probiotic taxa, indicating improved immunity and growth, showing that the microbiome is functionally adapted to salinity variations rather than merely a stress response [149]. Similarly, in *Coilia nasus*, salinity mitigation reduces harmful microbes, such as *Escherichia coli*, while enhancing good taxa such as *Actinobacteria* and *Corynebacterium*, which could be associated with improved health and stress tolerance under variable salinity conditions [150]. These studies indicate that microbiome shifts are more than just consequences of salinity stress; they may also play an active role in modifying the organism’s ability to withstand such stress.

Salinity stress induces species-specific shifts in gut microbiota that mechanistically compromise gut health, immune function, and nutrient absorption in aquatic organisms. In *Micropterus salmoides*, elevated salinity increases *Bacillus* abundance, which may enhance osmolyte production and stress tolerance [151]. In contrast, *Salmo salar* exhibits distinct microbial profiles in freshwater (*Firmicutes/Actinobacteria*) versus seawater (*Proteobacteria*), with the latter associated with pro-inflammatory responses through TLR/NF-κB signaling [152]. These microbial changes directly impact gut barrier integrity, as demonstrated in *Macrobrachium rosenbergii*, where hypotonic stress (≤8‰) promotes pathogenic *Vibrio* and *Flavobacterium* proliferation while reducing antioxidant capacity (↓SOD, ↑MDA/NO). This oxidative stress downregulates tight junction proteins and elevates iNOS activity, impairing intestinal barrier function and increasing susceptibility to inflammation [17]. Similarly, hypo-salinity in *Sebastes schlegelii* reduces beneficial *Photobacterium* (SCFA producers) while increasing *Mycoplasma*, leading to bile acid dysmetabolism and downregulation of nutrient transporters (SGLT-1/PepT1). These changes, coupled with suppressed NKA activity, disrupt ion-dependent nutrient absorption and exacerbate metabolic inefficiency [153].

**Figure 4:**
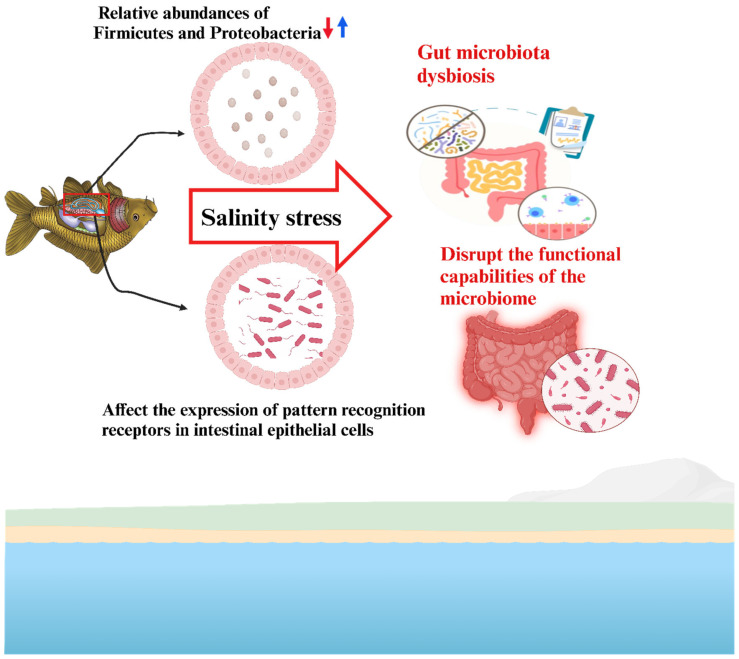
The impact of salinity stress on gut microbiota in fish, leading to dysbiosis, or imbalance in microbial communities. This dysbiosis can disrupt the functional capabilities of the microbiome, affecting the overall health and metabolic functions of the fish. The visual emphasizes the connection between environmental stressors and alterations in gut health.

In *L. vannamei*, hypoosmotic conditions led to Vibrio overgrowth, reduced microbial richness, and altered lipopolysaccharide biosynthesis [16]. Similarly, in mud crabs, salinity fluctuations increased intestinal permeability, promoted pathogen translocation, and activated inflammatory pathways, exacerbating microbial imbalance [46]. Figure 4 demonstrates how salinity stress induces changes in the gut microbiota of aquatic organisms, leading to gut microbiota dysbiosis. Under fluctuating salinity conditions, the microbial composition in the gut becomes imbalanced, with beneficial microbes potentially decreasing and less beneficial or harmful microbes increasing. This dysbiosis disrupts the functional capabilities of the microbiome, impairing essential processes such as nutrient absorption, immune system regulation, and overall gut health. The figure highlights that these disruptions can compromise the organism’s ability to adapt to salinity stress, potentially affecting its survival and physiological functions. Overall, this underscores the critical role of a balanced microbiome in maintaining the health and stress resilience of aquatic organisms exposed to changing salinity environments.

Functional studies reveal that the gut microbiota play an important role in fish osmoregulation via distinct pathways. In Medaka, microbial ecotone manufacturing in seawater-adapted fish was associated with increased Na^+^/K^+^-ATPase and cftr expression in gills following ecotone treatment, establishing a direct relationship between microbiota activity and osmoregulatory gene regulation. Under acute salinity stress, Nile tilapia exhibited microbial enrichment of osmoregulatory genes (myo-inositol-1-monophosphatase, glutathione S-transferase) and ABC transporters, while carbohydrate-active enzymes were depleted, indicating microbiota-mediated metabolic reprogramming for osmotic balance [154]. Transcriptomic investigations in teleosts validated microbiota’s influence on ion-transport genes and osmolyte production pathways during salinity adaptation [155]. These studies provide evidence that gut microbiota plays a functional role in osmoregulation by regulating gene expression and producing metabolites under salinity stress.

At the molecular level, salinity-induced microbiome shifts modulate the expression of stress response genes, antimicrobial peptides, and the prophenoloxidase system, thereby influencing how invertebrates maintain intestinal homeostasis [156]. Under low salinity, structural proteins such as mucins and peritrophins are upregulated, fortifying the intestinal barrier and favoring beneficial taxa [157]. However, hypoosmotic conditions also elevate oxidative stress markers, which can damage intestinal tissues and further disrupt host–microbiome interactions [158]. Thus, salinity stress significantly alters host–microbiome interactions by shifting the microbial community composition, disrupting metabolic functions, and compromising immune and intestinal homeostasis, ultimately impacting host health and stress tolerance.

Microbiome modifications owing to salinity stress in aquaculture can be efficiently addressed using probiotics or microbial consortia. The disturbance of gut microbiota under varied salinity conditions, resulting in lower species richness and increased disease susceptibility, can be addressed by introducing beneficial microbial communities. For example, microbial consortia containing ammonia-oxidizing bacteria, nitrite-oxidizing bacteria, and denitrifying bacteria have been shown to reduce total ammonia nitrogen levels in shrimp farms, improving water quality and reducing salinity stress [159]. Probiotics, on the other hand, have been shown to balance gut microbiota, boost immunological responses, and improve pathogen resistance in response to environmental stress [158]. These microbiome-based techniques provide a sustainable and cost-effective strategy to reduce salt stress, enhance shrimp health, and preserve productivity in aquaculture systems.

## 5. Genetic Diversity and Resilience of Aquatic Species

Genetic diversity is fundamental to aquatic organisms’ resistance to environmental stresses, which include both chemical contaminants and abiotic factors such as salinity changes. In the crab *Portunus armatus*, genetic traits impose tolerance to elevated temperatures and salinity changes [160], whereas in *Chironomus riparius*, populations exposed to long-term mercury contamination developed elevated mercury tolerance without genetic erosion, implying directional selection for resistant traits. In addition, in both situations, communities with greater baseline genetic diversity performed better under stress conditions [161]. These findings highlight the dual significance of genetic diversity as a reservoir for adaptive traits under directional selection and as a barrier against environmental obstacles [61].

However, extreme variations in salinity can negatively impact genetic diversity. For example, shifts in salinity have been linked to noticeable changes in the genetic structure of *Artemia* populations, leading to a decline in their diversity [162]. This highlights the negative impact that drastic environmental changes can have on genetic stability, which in turn affects the resilience of populations. Despite this, species with higher genetic diversity tend to fare better under fluctuating conditions, underlining the importance of maintaining genetic variation for adaptive capacity [163].

Salinity also influences population structure and gene flow within aquatic species. For example, the salinity barrier created by the Amazon-Orinoco plume causes genetic differences between Atlantic fiddler crabs populations from the Caribbean and Brazil, showing how environmental factors can separate populations and limit gene flow [164]. On the other hand, the more adaptable *Callinectes danae* exhibits less genetic differentiation and more gene flow, indicating its ability to better cope with salinity fluctuations [165]. This comparison highlights the differential adaptability of species to salinity fluctuations, emphasizing the subsequent effects on genetic diversity.

Hybridization studies on tilapia show that hybrid fish have faster growth rates and greater tolerance to salinity fluctuations compared to their purebred counterparts, demonstrating how hybridization can increase genetic diversity and improve the ability to cope with environmental stress [166]. Similarly, *F. heteroclitus* exhibit significant genetic variations in genes associated with environmental stress responses, such as the aryl hydrocarbon receptor, which enhance their ability to tolerate normally lethal levels of stress [137]. In addition, *Esox lucius* fish populations have evolved flexible reproductive strategies, with certain fish species able to produce more embryos in saline environments than their freshwater counterparts, further enhancing their ability to survive in variable habitats [167]. These reproductive adaptations are another way in which genetic diversity supports resilience in dynamic environments. Future research should focus on evaluating the long-term evolutionary impacts of hybridization on population genetics, particularly the persistence of hybrid vigor and the potential for genetic assimilation over multiple generations.

## 6. Strategies for Mitigating Salinity Stress in Aquatic Organisms

Effective strategies to mitigate salinity stress in aqua cultural systems include nutritional interventions and the development of salinity-tolerant strains.

### 6.1. Nutritional Intervention

Dietary adjustments play a key role in enhancing osmoregulatory efficiency and stress resilience. For example, in *L. vannamei* protein requirements increase under higher salinities to optimize growth and osmoregulatory functions [168]. Supplementing with essential amino acids, such as methionine and tryptophan, further supports physiological stability under salinity stress [169]. In addition to proteins, lipid metabolism also supports energy demands for osmoregulation. Supplementing the diet with lipids enhances energy availability and osmoregulatory functions in shrimps, especially when bile salt production is insufficient [170]. Furthermore, n-3 polyunsaturated fatty acids (PUFAs, e.g., EPA and DHA) not only enhance growth and immune responses in aquatic organisms, but also contribute to stress resilience by maintaining membrane fluidity, reducing oxidative damage, and modulating inflammatory pathways crucial for counteracting osmotic stress [171]. Other key nutrients such as myo-inositol, choline, and vitamin D3 improve antioxidative function, thereby boost salinity stress resilience in *L. vannamei* [170]. For instance, dietary supplementation of vitamins E and C enhanced growth performance and salinity tolerance in *L. vannamei* [172]. Long-term supplementation with nutrients including vitamins (vitamins C and E) and minerals (selenium) has been revealed to improve stress tolerance in aquatic animals, including higher osmoregulatory ability and immunological responses under chronic stress [173]. Using fish oil and soybean oil in the diet of mud crabs improves nutritional value, taste, and odor. Meanwhile, low salinity negatively affected taste and odor, indicating that the presence of fish oil might reduce these consequences [174]. Carotenoids, including astaxanthin, further enhance osmotic stress resistance in crustaceans, strengthening their ability to cope with salinity fluctuations [175]. Increased calcium levels shorten molt intervals and accelerate exoskeletal calcification, optimizing crab production [176]. Additionally, nucleotides supplementation has shown considerable effects on osmoregulatory competence and enhanced survival rates during salinity transitions in fish species such Atlantic salmon and red sea bream [177]. These results highlight the importance of a balanced diet, including proteins, lipids, vitamins, nucleotides, and minerals, in supporting osmoregulatory function and maintaining the health of aquatic species under fluctuating salinity conditions. In species like *O. niloticu*, salinity-induced morphological changes in the intestine can disrupt nutrient absorption, highlighting the need for a tailored amino acid profile to sustain protein digestibility [178]. Fish populations also require flexible reproductive strategies to thrive in saline environments. Supplementation with essential fatty acids, such as DHA, has been shown to improve salinity tolerance in fish larvae [179]. Furthermore, carbohydrates can act as protein-sparing agents under metabolic stress in fish, improving overall nutrient utilization. For example, diets containing 15–20% carbohydrates benefit fish in osmoregulatory processes [180].

In addition to traditional nutrients, commercial products such as Optimun (Chemoforma, Augst, Switzerland) have been found to reduce plasma cortisol and glucose levels in rainbow trout and red drum, thereby enhancing acute stress tolerance [181]. Vannagen™ (Chemoforma, Augst, Switzerland) has been shown to improve stress resilience in fish, with studies on sole confirming its efficacy in lowering stress-induced cortisol levels [182]. Another product, Maxi-Gen™ Plus Canadian Bio-Systems Inc. (Calgary, AB, Canada), has been successfully incorporated into the diets of Atlantic salmon to improve hypo-osmoregulatory ability and reduce cortisol levels during smoltification, thus increasing growth performance and stress resilience [183].

### 6.2. Development of Salinity-Resistant Varieties

Selective breeding has emerged as a promising strategy to enhance salinity tolerance in aquatic species. For instance, the Sukamandi strain of tilapia, a hybrid between *O. niloticus* and *O. aureus*, demonstrates increased salinity tolerance through gene introgression from *O. aureus*. Selective breeding for these traits has reinforced the salinity tolerance of this strain, making it better suited for environments with fluctuating salinity levels [184]. Similarly, selective breeding programs targeting *P. hypophthalmus* have successfully enhanced growth and survival in saline environments [185]. In shrimp aquaculture, the identification of genetic markers linked to salinity tolerance has facilitated the distinction between salinity-tolerant families and those more susceptible to environmental stress. This enables more efficient selection of brood stock with inherent salinity-resistant traits, improving the overall effectiveness of breeding programs aimed at enhancing resilience to salinity fluctuations [186].

However, intensive selective breeding for salinity tolerance entails risks that must be carefully analyzed. One important concern is the population’s low genetic diversity, which may hinder the species’ capacity to respond to other environmental stressors such as disease outbreaks or temperature variations [163]. Another major issue is inbreeding depression, which can cause decreases in vital traits including growth, reproduction, and overall fitness, rendering the population more vulnerable to various challenges [187]. Furthermore, intensive selective breeding may result in the loss of significant genetic features that are important for future breeding demands or in response to unexpected environmental changes [188]. These risks highlight the significance of balancing selective breeding efforts with techniques that preserve genetic diversity and ensure the population’s long-term resilience.

To implement strategies for mitigating salinity stress while supporting biodiversity preservation in natural habitats, it is essential to balance interventions with ecological sustainability. Nutritional interventions and selective breeding should be carefully managed to avoid disrupting natural food webs and genetic diversity. For instance, while nutrient supplementation can enhance osmoregulatory functions in aquaculture [174], similar approaches in natural ecosystems should use native, ecologically appropriate resources to support local species without introducing non-native elements. Selective breeding efforts aimed at enhancing salinity tolerance should be paired with genetic studies to preserve wild genetic diversity, preventing the risks of inbreeding and loss of adaptability.

## 7. Shortcomings

Despite significant progress in understanding the effects of salinity stress, several research gaps remain that hinder a more comprehensive understanding. Notably, there is a lack of detailed species-specific responses to both acute and chronic salinity fluctuations, which are essential for understanding how different species cope with salinity stress over both short-term and long-term periods. Insufficient long-term ecological studies limit our ability to predict cumulative effects of salinity stress over time, and there is limited knowledge regarding the interactive effects of salinity with other environmental stressors such as temperature and pollution. Additionally, the molecular mechanisms underlying behavioral adaptations to salinity changes remain underexplored, and there is a lack of comparative data on ion-transport mechanisms across diverse species.

The time-dependent nature of salinity stress is especially important, as short-term exposure may lead to acute physiological responses, while long-term stress can trigger chronic adaptations, potentially altering species fitness and survival. Further investigation into the long-term effects and heritability of epigenetic changes in response to salinity stress is needed to assess their potential for transgenerational adaptation. Finally, the decline in genetic diversity, especially in the context of rapid climate change, calls for increased research into how genetic variation can influence species’ ability to adapt to environmental changes. Addressing these shortcomings is vital for developing a more comprehensive understanding of salinity stress and for implementing effective strategies to protect aquatic ecosystems amid increasing environmental variability.

## 8. Conclusions

Salinity stress is a significant environmental factor that shapes aquatic ecosystems, influencing species distribution and altering biodiversity. The ability of species to adapt to fluctuations in salinity levels through diverse physiological, behavioral, and molecular mechanisms is crucial for maintaining ecosystem stability and resilience. Genetic diversity plays a key role in allowing populations to cope with changing salinity, ensuring long-term survival and adaptability. Furthermore, the intricate relationships between aquatic organisms and their microbiomes under salinity stress underscore the importance of microbial balance for overall health and resilience. Strategies such as nutritional interventions and the selective breeding of salinity-resistant strains show promise for enhancing the tolerance of aquaculture species to salinity stress, which also has implications for preserving biodiversity in natural habitats.

## Figures and Tables

**Table 2:** Key pathways involved in salinity stress response in aquatic organisms.

Specie	Pathway	Salinity Level	Duration	Effects	References
*Oreochromis niloticus*	AMPK	8 and 16 psu	8 weeks	Mediates energy metabolism under stress, maintaining cellular energy homeostasis.	[96]
*Rachycentron canadum*	MAPK	Acute stress (30 ppt to 10 ppt for one hour), Chronic (30 ppt–10 ppt for 4 days)	1 h to 28 days	MAPK pathway upregulated under acute salinity stress, inducing apoptosis and inflammation; chronic stress shows varied expression for long-term adaptation.	[97]
*Ictalurus punctatus*	MAPK	3 and 7 psu	3 weeks	p38-MAPK mediates gene expression, metabolism, and cellular homeostasis under salinity stress.	[98]
*Takifugu fasciatus*	MAPK	0, 10, and 20 ppt	3 days	20 ppt salinity led to increased oxidative stress, and apoptosis-related gene expression.	[99]
*Scylla paramamosain*	AMPK	23 and 4 ppt	6 weeks	Regulates stress responses, gene expression, apoptosis, and oxidative stress management; protects against cell damage via p38-MAPK.	[100]
*Scophthalmus maximus*	PI3K-AKT	5, 10, and 30 ppt	24 h	Low saline stress downregulated gene expression, protein content, and AKT1 phosphorylation in gill tissues.	[101]
*Litopenaeus vannamei*	AMPK	3, 20, and 30 psu	0 h to 96 h	AMPK elevated during acute stress, indicating higher energy needs; sustained during chronic stress for osmoregulation and energy balance.	[102]
*Litopenaeus vannamei*	RAS	25 and 3 ppt	Short-term stress (0 h–96 h), Long-term stress (8 weeks)	RAS pathway downregulated under stress, affecting homeostasis, osmoregulation, and blood glucose levels.	[103]
*Scylla paramamosain*	RAS	3, 20, and 23 ppt	0 h to 168 h	NKA activity increased under stress to regulate osmotic balance, aided by RAS pathway for improved metabolic activity.	[104]
*Penaeus monodon*	PI3K-AKT	30, 18, and 3 psu	45 days	Prolonged low saline stress downregulated RAS pathway, diverting energy from growth to ionic homeostasis and osmoregulation.	[105]

## Data Availability

No new data were created or analyzed in this study.
